# First dosimetric evaluation of clinical raster-scanned proton, helium and carbon ion treatment plan delivery during simultaneous real-time magnetic resonance imaging

**DOI:** 10.1016/j.phro.2025.100722

**Published:** 2025-02-05

**Authors:** Sebastian Klüter, Karolin Milewski, Wibke Johnen, Stephan Brons, Jakob Naumann, Stefan Dorsch, Cedric Beyer, Katharina Paul, Kilian A. Dietrich, Tanja Platt, Jürgen Debus, Julia Bauer

**Affiliations:** a Department of Radiation Oncology, Heidelberg University Hospital, Heidelberg, Baden-Württemberg, Germany; b Heidelberg Institute of Radiation Oncology (HIRO), National Center for Radiation Oncology (NCRO), Heidelberg, Baden-Württemberg, Germany; c Medical Physics in Radiation Oncology, German Cancer Research Center (DKFZ) Heidelberg, Heidelberg, Germany; d Heidelberg Ion-Beam Therapy Center (HIT), Heidelberg, Baden-Württemberg, Germany; e Medical Physics in Radiology, German Cancer Research Center (DKFZ) Heidelberg, Heidelberg, Germany; f National Center for Tumor Diseases (NCT), Heidelberg, Baden-Württemberg, Germany; g German Cancer Consortium (DKTK), Core-center Heidelberg, Heidelberg, Baden-Württemberg, Germany; h Clinical Cooperation Unit Radiation Oncology, German Cancer Research Center (DKFZ) Heidelberg, Heidelberg, Baden-Württemberg, Germany

**Keywords:** MR-integrated particle therapy, Online MR-guidance, In-beam MR imaging, MRI, Proton, Helium, Carbon ions

## Abstract

This work presents an experimental dosimetric evaluation of raster-scanning particle beam delivery during simultaneous in-beam magnetic resonance (MR) imaging. Using an open MR scanner at an experimental treatment room, radiochromic film comparisons for protons, helium and carbon ions, each with and without simultaneous in-beam cine MR imaging, yielded 2D gamma pass rates ≥ 98.8 % for a 3 % / 1.5 mm criterion, and ≥ 99.9 % for 5 % / 1.5 mm. These results constitute a first experimental confirmation that time varying magnetic fields of MR gradients do not result in clinically relevant additional dose perturbations.

## Highlights

• Demonstration of ion beam delivery with in-beam magnetic resonance imaging (MRI).
• Film analyses show that simultaneous MRI does not distort ion dose distributions.
• Raster scanning ion beam delivery can be combined with real-time in-beam MRI.
• Shown for clinical proton, helium and carbon ion treatment plans.

## 1 Introduction

It is well known that in particle therapy (PT), anatomical changes can lead to significant dose deviations due to the sharp dose fall off behind the Bragg Peak [1], [2]. The integration of Magnetic Resonance (MR) imaging at the treatment isocenter therefore holds great potential for increasing the accuracy of PT [3] and has gained more and more interest recently [4]. Besides the superior soft-tissue contrast [5] that could be exploited for adaptive PT [6], integrated MR imaging (MRI) would also enable gating and tracking based on internal anatomy, like already clinically implemented with MR-Linacs in photon radiotherapy [7]. In addition, first reports indicate that online MRI could also be used for visualization of the Bragg peak in water, potentially introducing new methods for particle range verification [8].

While the benefits are apparent and the conceptual feasibility has been shown [9], many technical obstacles concerning the integration of pencil beam scanning particle beam delivery and MRI at the treatment isocenter still need to be solved before treatment of the first patients [10]. Since both active beam scanning and MRI apply time-varying magnetic fields, their mutual interference needs to be thoroughly evaluated. On the imaging side, it has been shown that the fringe field of the beam scanning magnets can produce artifacts in simultaneously acquired MR images [11], [12].

On the beam delivery side, deflection and distortion of the particle beam due to the static magnetic field of the MR scanner have been described [13], [14], [15], and, although no commercial solutions ready for clinical implementation exist yet, these effects can in principle be accounted for by means of treatment planning [16]. During simultaneous irradiation and MRI, not only the static magnetic field, but also the rapidly switching gradient fields act on the particle beam. In a simulation study, Santos et al. [17] have shown that gradient fields can result in a blurring effect on an infinitely thin proton beam during simultaneous in-beam MRI, but expected no clinically relevant dosimetry implications for realistic beam widths. However, no experimental dosimetric validations of this hypothesis have yet been reported.

A 0.25 T open MR scanner was installed onto a movable platform inside an experimental ion beam therapy treatment room to enable in-beam MRI. The purpose of the present work was to dosimetrically evaluate clinical ion beam treatment plan delivery using active raster scanning during simultaneous in-beam MRI.

## 2 Materials and method

### 2.1 In-beam MRI and receive coil

An open orthopedic MR scanner with a C-shaped permanent magnet with a vertical magnetic field of 0.25 T (Esaote S-Scan, Esaote, Italy) was installed together with a radiofrequency (RF) shielding cabin on a movable platform in the experimental irradiation room at the Heidelberg Ion beam Therapy center (HIT) [12]. The gradient system of the scanner has a nominal maximal strength of 20 mT/m and a nominal maximal slew rate of 56 T/m/s. Horizontal beam entrance into the cabin and thus into the imaging field-of-view was possible through a 20 x 20 cm^2^ copper foil window (see Ref. [12] for a more detailed description of the setup).

In-beam imaging was performed using an in-house developed solenoid RF receiver coil for extremities [18], consisting of a polymethyl methacrylate (PMMA) cylinder with a diameter of 18 cm and a length of 15 cm, onto which a flat, 35 µm thick copper conductor, embedded between two layers of flexible polyimide and acrylic-based adhesive, was glued using the same adhesive (see Supplementary Fig. S1). This design was previously used for a transmit and receive RF body coil integrated with a patient rotation system for MR-integrated particle therapy (MRiPT) [19]. With a resulting difference in water equivalent thickness between conducting and non-conducting coil parts of only 210 µm, the design allows for particle irradiation through the coil (see Ref. [19] for more details).

For simultaneous MRI during irradiation, a transversally oriented 2D balanced Steady State Free Precession (bSSFP) cine pulse sequence was used. The sequence parameters were manually optimized for image quality in the phantom (TR = 8 ms, TE = 4 ms, frame rate = 0.51 fps, 1.25 x 1.49 mm^2^ pixel size, 14 mm slice thickness).

### 2.2 Phantom and setup

A modular cylindrical phantom was designed in order to allow dose measurement with radiochromic films at variable depths while also providing sufficient signal for MRI. It consists of several disc-shaped PMMA segments, either solid or filled with water. Between each two discs, a gap for placement of round Gafchromic EBT3 film cut-outs (Ashland, USA) was foreseen. An outer diameter of 10 cm was chosen for the phantom so that it would fit into a commercial knee-coil as well as into the in-house developed extremity coil. Films were positioned at physical depths of 1.0 cm, 6.9 cm and 10.9 cm in the phantom (see Supplementary Fig. S1).

The phantom was positioned inside the coil using a 3D-printed holder. Longitudinally, the coil was centered in the MR isocenter, and the phantom surface was aligned with the coil edge. Laterally, the phantom was shifted by 3 cm for the proton field and by 1.5 cm for the helium field due to dose displacements caused by the static magnetic field of the MR scanner. Irradiations were performed along the cylinder axis of the coil (see Supplementary Fig. S1).

### 2.3 Plan measurements

For proton, helium and carbon ion irradiation, one field each was copied from clinical treatment plans and applied to the phantom using active raster-scanning [20] (see Supplementary Table S1 for more details on the treatment fields). Dose for all fields was scaled to yield roughly 2 Gy in the high dose region.

All irradiations were performed inside the MR scanner, once without and once with simultaneous imaging for each field. Films were digitized after 36 h and calibrated using particle-specific monoenergetic calibrations. For line profile comparisons, films were aligned using handmarked external laser positions. For 2D gamma comparisons, films were further aligned automatically in order to exclude marking-errors, and gamma distributions were calculated using 3 % / 1.5 mm and 5 % / 1.5 mm criteria.

The acquired MR images of all phantom irradiations were used for monitoring of the phantom position in the x/y-plane perpendicular to the beam entry axis.

## 3 Results

**Fig. 1:**
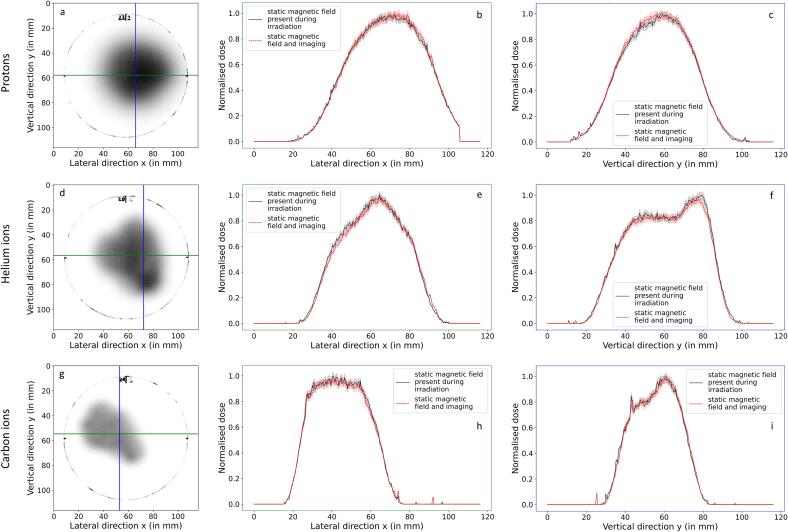
Dose-calibrated Gafchromic EBT3 films irradiated with simultaneous MR imaging in a phantom depth of 6.9 cm for the proton plan (a), helium ion plan (d) and carbon ion plan (g). Additionally, line profile comparisons between irradiations with and without simultaneous imaging are shown in horizontal (b,e,h) and vertical (c,f,i) film directions. Profiles for the irradiations without imaging are shown in black, profiles for the irradiations during simultaneous MR imaging are shown in red, both together with a 3 %-wide shaded area in order to better visualize agreement within 3 %. (For interpretation of the references to colour in this figure legend, the reader is referred to the web version of this article.)

Fig. 1 exemplary shows line profile comparisons between films irradiated at a phantom depth of 6.9 cm with and without simultaneous MRI for the delivered proton, helium and carbon ion treatment field. It can be seen that the dose agreement in the high dose regions lies well within 3 % for all profiles. More importantly, no relevant shifts of the dose distributions are visible for the two scenarios. All line profiles in the other depths (not shown) compared equally well.

**Fig. 2:**
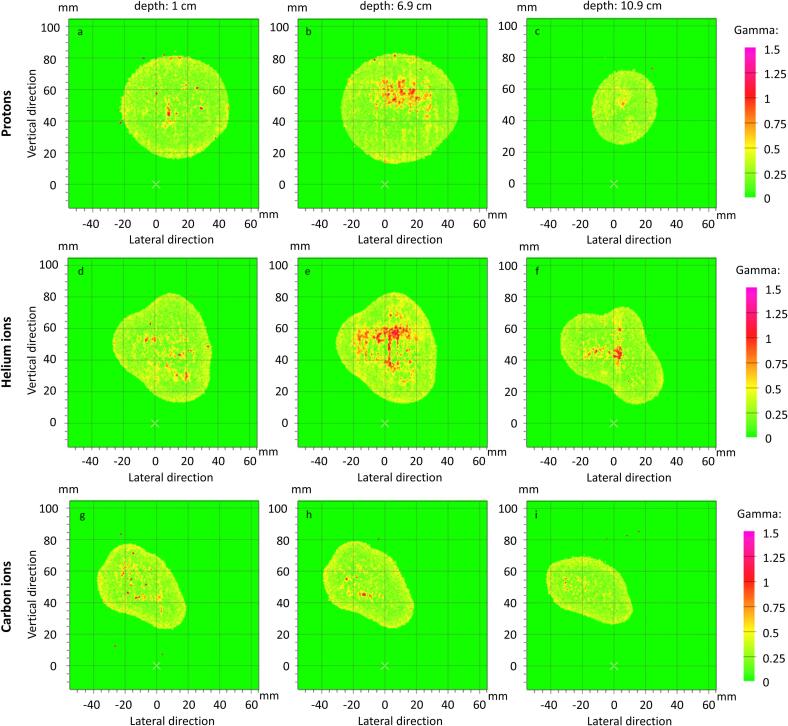
Results of 2D gamma comparisons (3 % / 1.5 mm) between films irradiated with and without simultaneous MR imaging in phantom depths of 1 cm (a,d,g), 6.9 cm (b,e,h) and 10.9 cm (c,f,i), for protons (a,b,c)), helium ions (d,e,f) and carbon ions (g,h,i).

2D gamma comparisons (3 % / 1.5 mm) for all depths are depicted in Fig. 2. The lowest pass rate was 98.8 %, all others were ≥ 99.5 %. With a criterion of 5 % / 1.5 mm, which is clinically used at HIT, all pass rates were ≥ 99.9 % (see Supplementary Table S2).

## 4 Discussion

In this study, clinical ion beam treatment fields were delivered using active raster-scanning with simultaneous in-beam MRI for the first time. The presented dosimetric evaluations of films in different measurement depths prove that simultaneous MRI does not alter clinical dose distributions for ions compared to irradiation inside the static magnetic field of the MR scanner but without imaging.

Dose was measured using radiochromic films, which lead to a slightly larger measurement uncertainty than compared to for example ionization chambers. While it has been shown that ion chamber dosimetry indeed is feasible for protons and carbon ions in the presence of magnetic fields [21], [22], [23], films were chosen for three reasons. First, since only small dose variations were expected, a high resolution was needed; point dose measurements as performed on MR-Linacs using ionization chambers or plastic scintillators [24] were deemed pointless in our case. Second, dose measurement during simultaneous MRI is not trivial, since devices containing electronics could potentially be damaged due to induction of currents. Third, in order to produce sufficient signal for MRI, a phantom filled with water or other liquid was necessary, which further complicated phantom design.

The effects of the static magnetic field of an MR scanner on charged particles in MRiPT have been evaluated by various authors. Depending on the orientation of the beam to the magnetic field, the Lorentz force either causes a deflection of the beam and distortion of the spot shape, or a rotation of the treatment field [3], [13], [14], [15], [25], [26]. In our case, the lateral displacement due to the static magnetic field was taken into account by shifted phantom positions. Dose calculation algorithms for charged particles that are able to take the static magnetic field into account have been developed [16], [26], [27]. It has also been shown that the effects can be adequately corrected through adapted treatment plan optimization [26], [28]. Future studies should extend the present work to a full validation from treatment planning and dose calculation in the magnetic field up to irradiation with simultaneous MRI. This should include inhomogeneous phantoms [15] as well as larger phantoms in order to cover the full clinically available treatment area.

Potential effects of the dynamic fields of an MR scanner’s gradient system on the treatment beam have been evaluated through simulations by Santos et al. [17]. The authors described a blurring effect on an infinitely thin pencil beam source caused by imaging gradients of a bSSFP pulse sequence with either 20 mT/m or 40 mT/m maximum gradient strength. This effect was more pronounced for the stronger gradient strength, but rapidly became less significant with increasing beam width. Using a clinically more realistic beam width of σ = 3 mm, they concluded no clinical dosimetry implications.

The maximum additional deflection for a perpendicular static field gradient of 20 mT/m would in our setup amount to roughly 0.3 mm for the proton plan and even less for the helium and carbon ion plans within an irradiation field of 20 cm x 20 cm. While time-varying imaging gradients acting in 3D lead to a blurring effect rather than a mere deflection, the order of magnitude is still valid. Thus, no relevant effects were to be expected, which we could confirm.

In our setup, the nozzle was placed outside the RF cabin, excluding any RF-effects due to simultaneous imaging on the nozzle detectors. In addition to RF, also the magnetic field of the MR scanner and acoustic noise could influence the nozzle detectors during simultaneous MRI, therefore MR-compatible nozzle detectors are desirable for MRiPT [29], [30]. On the other hand, it has also been shown that the magnetic fringe fields of the beam scanning magnets can impact image quality during simultaneous MRI [11]. However, these effects are dependent on the specific timing of the applied beam scanning, the distance between the beam scanning magnets and the MR isocenter [12] as well as the used MR pulse sequence. First results indicate that the effects might be negligible for 2D bSSFP imaging [12].

The acquired cine MR images allowed monitoring of the phantom position during irradiations, which is somehow trivial for the static case. In future studies, it is planned to use a moving phantom in order to further move towards beam gating based on online MRI for MRiPT. The imaging framerate and slice thickness used in the present study yielded the best image quality for the phantom with given limitations of the used orthopedic MR scanner, but will need to be improved in future gating experiments. At MR-Linacs, typically up to 8 fps and slice thicknesses of 5 mm or 7 mm are clinically used for MRI-based gating [31], [32], [33].

In conclusion, simultaneous MRI and clinical treatment plan delivery using actively raster-scanned proton, helium and carbon ion beams was demonstrated for the first time. This concludes an important step towards MRiPT. For the clinical introduction, further experiments will need to extend our results to faster framerates of real-time MRI, larger treatment fields and also next generation MR scanners.

## Funding information

This work was kindly sponsored by the German Federal Ministry of Education and Research within the funding program “Bildgeführte Diagnostik und Therapie—Neue Wege in der Intervention” (ARTEMIS, 13GW0436A). For the publication fee we acknowledge financial support by Heidelberg University.

## CRediT authorship contribution statement

**Sebastian Klüter:** Conceptualization, Formal analysis, Funding acquisition, Investigation, Methodology, Writing – original draft, Writing – review and editing. **Karolin Milewski:** Conceptualization, Formal analysis, Investigation, Methodology, Writing – review and editing. **Wibke Johnen:** Investigation, Writing – review and editing. **Stephan Brons:** Investigation, Methodology. **Jakob Naumann:** Conceptualization, Formal analysis, Methodology, Writing – review and editing. **Stefan Dorsch:** Investigation, Methodology. **Cedric Beyer:** Investigation, Methodology. **Katharina Paul:** Conceptualization, Methodology. **Kilian Dietrich:** Investigation, Methodology, Writing – review and editing. **Tanja Platt:** Investigation, Methodology, Writing – review and editing. **Jürgen Debus:** Funding acquisition, Supervision, Resources. **Julia Bauer:** Conceptualization, Formal analysis, Investigation, Methodology, Writing – review and editing.

## Declaration of competing interest

The authors declare the following financial interests/personal relationships which may be considered as potential competing interests: SK received speaker fees from Siemens Healthineers. JD received grants from View Ray Inc., CRI—The Clinical Research Institute GmbH, Accuray Incorporated, Accuray International, RaySearch Laboratories AB, Vision RT limited, Astellas Pharma GmbH, Astra Zeneca GmbH, Solution Akademie GmbH, Ergomed PLC Surrey Research Park, Merck Serono GmbH, Siemens Healthcare GmbH, Quintiles GmbH, Pharmaceutical Research Associates GmbH, Boehringer Ingelheim Pharma GmbH Co, PTW-Freiburg GmbH, Nanobiotix A.A. and IntraOP Medical, all outside the submitted work.
