# Supplementary material for: First dosimetric evaluation of clinical raster-scanned proton, helium and carbon ion treatment plan delivery during simultaneous real-time magnetic resonance imaging
Source: Phys Imaging Radiat Oncol. 2025 Feb 5;33:100722. doi: 10.1016/j.phro.2025.100722 (PMC11870259; doi:10.1016/j.phro.2025.100722)
Supplement: Supplementary Data 1 [file mmc1.pdf]

Supplementary Material

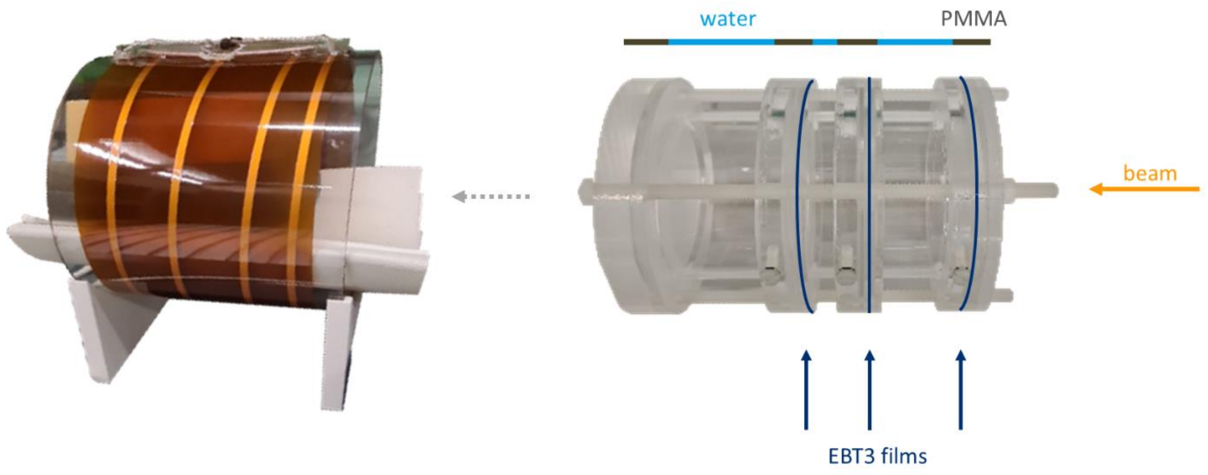

Figure S1: In-house developed RF receive coil (left) for the in-beam MRI as well as in-house developed cylindrical phantom for film measurement of clinical particle treatment plans with simultaneous MR imaging. The modular phantom is constructed of discs of different thicknesses filled alternately with water and solid PMMA and sheets of Gafchromic EBT3 films in between, the blue arrows show the film positions at depths of 1.0 cm, 6.9 cm and 10.9 cm. The phantom was positioned inside the coil, beam entrance from the right is depicted by the orange arrow.

Table S1: Parameters of the individual fields of clinical treatment plans that were used for the film measurements in the cylindrical phantom with and without simultaneous MR imaging. All fields were delivered using active raster-scanning.

	Protons	Helium ions	Carbon ions
Clinical treatment	Meningioma	Astrocytoma	Chondrosarcoma
# Iso-energy layers	21	17	15
Min. beam energy [MeV/u]	91.48	107.24	212.12
Max. beam energy [MeV/u]	131.71	137.28	262.7
Spot sizes (FWHM) in water [mm]	12.4-17.2	7.2-8.9	6.5-8.2

Table S2: Results of 2D gamma comparisons between films irradiated with and without simultaneous MR imaging in different phantom depths, using gamma criteria of 3 % / 1.5 mm and 5 % / 1.5 mm, (the latter is the clinically used criterion at the Heidelberg Ion Beam Facility).

		Protons [%]	Helium ions [%]	Carbon ions [%]
Gamma 3% / 1.5 mm				
Depth	1 cm	99.7	99.8	99.7
	6.9 cm	99.7	98.8	99.8
	10.9 cm	100.0	99.5	99.9
Gamma 5% / 1.5 mm				
Depth	1 cm	99.9	99.9	99.9
	6.9 cm	99.9	99.9	99.9
	10.9 cm	100.0	99.9	100.0
